# Investigation of the Role of PUFA Metabolism in Breast Cancer Using a Rank-Based Random Forest Algorithm

**DOI:** 10.3390/cancers14194663

**Published:** 2022-09-25

**Authors:** Mariia V. Guryleva, Dmitry D. Penzar, Dmitry V. Chistyakov, Andrey A. Mironov, Alexander V. Favorov, Marina G. Sergeeva

**Affiliations:** 1 Faculty of Bioengineering and Bioinformatics, Lomonosov Moscow State University, 119234 Moscow, Russia; 2 Vavilov Institute of General Genetics, Russian Academy of Sciences, 119991 Moscow, Russia; 3 Belozersky Institute of Physico-Chemical Biology, Lomonosov Moscow State University, 119992 Moscow, Russia; 4 Kharkevich Institute of Information Transmission Problems, Russian Academy of Sciences, 127051 Moscow, Russia; 5 School of Medicine, Johns Hopkins University, Baltimore, MD 21218, USA

**Keywords:** breast cancer, machine learning, PUFAs, transcriptomics, random forest

## Abstract

**Simple Summary:**

Polyunsaturated fatty acids (PUFAs) and their derivatives, oxylipins, are a constant focus of cancer research due to the relationship between cancer and processes of energy metabolism and inflammation, where a PUFA system is an active player. Only recently have methods been developed that allow for studying such complex systems. Using the Rank-based Random Forest (RF) model, we show that PUFA metabolism genes are critical for the pathogenesis of breast cancer (BC); BC subtypes differ in PUFA metabolism gene expression. The enrichment of BC subtypes with various genes associated with oxylipin signaling pathways indicates a different contribution of these compounds to the biology of subtypes.

**Abstract:**

Polyunsaturated fatty acid (PUFA) metabolism is currently a focus in cancer research due to PUFAs functioning as structural components of the membrane matrix, as fuel sources for energy production, and as sources of secondary messengers, so called oxylipins, important players of inflammatory processes. Although breast cancer (BC) is the leading cause of cancer death among women worldwide, no systematic study of PUFA metabolism as a system of interrelated processes in this disease has been carried out. Here, we implemented a Boruta-based feature selection algorithm to determine the list of most important PUFA metabolism genes altered in breast cancer tissues compared with in normal tissues. A rank-based Random Forest (RF) model was built on the selected gene list (33 genes) and applied to predict the cancer phenotype to ascertain the PUFA genes involved in cancerogenesis. It showed high-performance of dichotomic classification (balanced accuracy of 0.94, ROC AUC 0.99) We also retrieved a list of the important PUFA genes (46 genes) that differed between molecular subtypes at the level of breast cancer molecular subtypes. The balanced accuracy of the classification model built on the specified genes was 0.82, while the ROC AUC for the sensitivity analysis was 0.85. Specific patterns of PUFA metabolic changes were obtained for each molecular subtype of breast cancer. These results show evidence that (1) PUFA metabolism genes are critical for the pathogenesis of breast cancer; (2) BC subtypes differ in PUFA metabolism genes expression; and (3) the lists of genes selected in the models are enriched with genes involved in the metabolism of signaling lipids.

## 1. Introduction

Breast cancer (BC) is the leading cause of cancer death among women worldwide [1]. BC is a heterogeneous disease; such a feature determines the risk of disease progression and its resistance to therapy [2,3]. There are five molecular subtypes of BC: luminal A, luminal B, HER2-enriched, basal-like, and normal-like [4]. Depending on the subtype, various molecular mechanisms of the pathogenesis of neoplasia are realized [3], while the patterns of metabolic phenotypes of molecular subtypes remain insufficiently studied [5].

The understanding of the relationship between changes in metabolism and cancer development has changed the focus of research several times over past decades [6]. Initially, studies of the mechanism of the “Warburg effect”, i.e., a metabolic switch from oxidative to glycolytic metabolism, attracted the greatest interest and, then, its relationship with the metabolism of nucleotides, lipids, and proteins [7]. Currently, metabolic rewiring has been recognized as an important feature to the progression of cancer in light of producing signal molecules [8]. 

One of the leading roles in the synthesis of signal lipid mediators is ascribed to the polyunsaturated fatty acids (PUFAs). Polyunsaturated fatty acids have more than one double bond in the carbon skeleton and represent a part of fatty acid (FA) metabolism. These acids are divided according to the double-bond position, the main important ones of which are the so-called Omega-3 (e.g., DHA and EPA) and Omega-6 (e.g., AA). Besides being structural components of the membranes and fuel sources for energy production, PUFAs also have a signaling function themselves or via their oxidative derivatives [9]. Both PUFAs and their oxidized derivatives, oxylipins, modulate the intrinsic cell programs and are utilized for communication with neighbor cells [10,11]. The important role of PUFAs and their corresponding oxylipins is an involvement in regulation of inflammatory processes. Omega-3 PUFAs (DHA and EPA) are attributed mainly to anti-inflammatory effects, while Omega-6 PUFAs, such as AA, are thought to be a part of proinflammatory pathways [12]. Oxylipins could be derived from Omega-3 as well as Omega-6 PUFAs [13,14]. Along with their precursors, oxylipins are responsible for inflammation and its subsequent resolution [15,16,17]. 

Inflammation is designated as a characteristic among the hallmarks of cancer [6,9,18,19], and unresolved, chronic inflammation, characterized by abnormal oxylipins synthesis, becomes fertile soil for malignant transformation and tumor immune evasion, including colorectal [12], gastrointestinal [20], colon [21,22], breast [23,24], pancreatic [25], prostate, and lung [26] cancers, and in melanoma [27]. Although for decades, it was known that the action of oxylipins is complex and is the result of PUFA metabolism through various enzymatic pathways [13,14,28], only recently did the development of omics technologies and algorithms for analyzing the data open up new opportunities in studying the role of PUFAs and their metabolites in the development of various diseases. Although there is evidence that individual oxylipins or the expression of genes responsible for their metabolism may be characteristics of different subtypes of BC [29,30], no systematic study of PUFA metabolism as a system of interrelated processes in this disease has been carried out. Further research concerning the role of dysregulated PUFA metabolism in the pathobiology of cancer holds great promise in uncovering novel metabolic and signaling nodes for targeted therapies.

Transcriptome analysis is one of the productive ways to study metabolic pathways alteration. It was shown that cancer metabolic reprogramming is regulated on the transcription level [31,32,33]. The heterogeneity of breast cancer and the variability of metabolic processes accompanying this heterogeneity requires a big amount of data to study. The data have been accumulated with the rise in next-generation sequencing (NGS) techniques and microarrays [34,35]. The joint analysis of such datasets via machine learning (ML) approaches could better resolve PUFAs’ roles in breast cancer development. 

ML approaches, particularly, the Random Forest (RF) [36] algorithm, have already been successfully applied to expression data analysis for various cancer types [37,38]. Nonetheless, the use of the entire data set is restricted by the differences in the corresponding technologies. Nonparametric methods independent of monotonic normalization can be used to overcome these limits [39,40]. Combining the nonparametric techniques, particularly, the ranking of the expression profile, with Random Forest simplifies the RF application on heterogeneous datasets. RF approaches are effectively implemented to distinguish heterogeneous groups, while, as far as we know, they have not been run for PUFA pathway analyses. 

In this study, we used an approach based on the combination of nonparametric method and RF model. Note that the Boruta feature selection [41,42] and Sequential Feature Selector [43,44] methods have been widely used and shown to be beneficial. The use of the Boruta feature selection algorithm [45] made it possible to identify the genes responsible for PUFA metabolism alterations in BC. The prediction ability of the classifiers was tested on independent datasets. The study has expanded our knowledge about the role of PUFA metabolic pathways in breast cancer pathogenesis and allowed us to identify their specific patterns in different molecular subtypes of breast cancer.

## 2. Materials and Methods

### 2.1. Data Source 

Transcriptome profiles for samples of breast cancer and normal adjacent tissues were used to train and validate the Random Forest model. For dichotomic classification of breast cancer and normal tissue samples, four datasets from the database Gene Expression Omnibus (GEO) were obtained (GSE65216, GSE29044, GSE1078, and GSE62944) (GEO; http://www.ncbi.nlm.nih.gov/geo/ (accessed on 18 February 2022)) (Supplementary Table S2). Dataset GSE62944 presents TCGA data from the TCGA (The Cancer Genome Atlas) database (Broad GDAC, https://gdac.broadinstitute.org/ (accessed on 18 February 2022)). Three datasets were used (GSE65216, GSE29044, and GSE1078) for the training set and included 221 tumor and 185 normal samples. TCGA data were used for the validation set and consisted of 1082 tumor and 113 normal samples. In order to distinguish among the subtypes of breast cancer by the differences in PUFA metabolism gene expression between five datasets (GSE81538, GSE25066, GSE31448, GSE96058, and GSE21653) with molecular subtypes, annotations were extracted from the GEO database (GEO; http://www.ncbi.nlm.nih.gov/geo/ (accessed on 18 February 2022)) (Supplementary Table S3).

### 2.2. Random Forest Model 

A Random Forest [36] predictor was built using the random Forest [46] R package. Briefly, Random Forest is an assembly of decision trees. It represents the union of two methods: bagging and random subspace method (RSM) [47]. Three main steps can be highlighted when building a Random Forest classifier:

1. From input data N × M (where N is the number of samples and M is the number of used features), k subsets are randomly selected with a return;
2. A decision tree is built for each subset;
3. The final decision is made on the majority vote for classification tasks or by averaging in the regression tasks.

Each decision tree includes a number of comparisons of the feature values and threshold, which is set during the model training. This fact limits the usage of random forest to gene expression data. In order to overcome this limitation, we ranked genes within a sample in both the training and test sets. 

After performing a ranking procedure, genes from the PUFA list presented in both the training and test sets (for dichotomic and multi-class classification separately) were selected. The Boruta algorithm (Boruta R package) was implemented on the training sets with the extracted PUFA genes (185 genes for tumor vs. normal samples predictor; 155 genes for molecular subtypes predictor) to shrink the number of studied genes. Final classifiers were built on the genes highlighted by Boruta as important ones (33 for tumor vs. normal samples comparison; 46 genes for molecular subtypes comparison) with the number of trees being 450 (Supplementary Figure S2). The code for the described analysis can be found at the GitHub repository: https://github.com/gurylevamv/PUFA_rRF (accessed on 21 September 2022).

### 2.3. Boruta Feature Selection Algorithm 

The feature selection algorithm Boruta was used to computationally identify the genes for which expression is important to distinguish between biological conditions. The main idea of this algorithm is to compare the features’ importance with the randomized version of themselves. The randomized features are referred to as shadows. Technically, a shadow feature is obtained from the initial one through value shuffling in a dataset copy. Two datasets, the initial features and the one with shadow features, are then merged. The Random Forest classifier is built on the merged dataset, and the importance of all features are calculated by the classifier. If the importance of an initial feature is greater than the maximum of shadow feature importance, then it receives 1 score. These operations are repeated a pre-given number (say N) of times. As a result, we obtain the sums of the scores for each feature after N trials. In a null model, these trials are binomially distributed. If the score of a feature is greater than the 99.5% quantile of the distribution, the feature is accepted as the important one. We used the Boruta algorithm as R package Boruta (accessed on 18 February 2022) [45]. The Boruta algorithm was applied to the PUFA gene list both for dichotomic (tumor vs. normal controls) and multi-class classifications. For greater confidence in the selected features, Boruta’s algorithm with the default cycles’ number parameter N (250) was run 100 times. The genes selected in 90% or more of the runs were finally considered as important genes. In the dichotomic sample comparison, 33 PUFA genes were selected by Boruta as important for classification. In the molecular subtype classification, 46 genes were selected as important.

### 2.4. Sequential Feature Selector for Minimal Gene Set Selection 

The Sequential Feature Selector (SFS) [48] was implemented to reach the minimal set of genes that shows the highest classification performance. This algorithm consequently selects a feature that will maximize the quality criterion function from the space of all features. Additionally, we used a floating extension of the SFS method (SFFS) that allows us to remove features if this step will make the prediction better. SFFS was used from the mlxtend Python package (accessed on 18 February 2022) [49].

### 2.5. SHAP Values to Identify the Most Important PUFA Genes 

SHAP refers to Shapley additive explanations, which is an approach that allows us to reach an explanation for the machine learning models output. It calculates the importance for each feature in each single sample. By applying SHAP values to the built predictors, we enhance their transparency, and moreover, in multi-class classification, it allows us to reach the importance of the feature in the separation of each class. SHAP calculations were performed via the SHAP package in Python (https://shap.readthedocs.io/ (accessed on 18 February 2022)).

### 2.6. Enrichment Analysis 

A GO functional annotation (Biological process, Molecular function) [50], and a KEGG [51] and Wiki pathway [52] enrichment analysis for the important PUFA genes revealed was performed via the Enrichr tool wrapped in python in the GSEApy package (https://gseapy.readthedocs.io/en/latest/introduction.html#gseapy-enrichr-module (accessed on 18 February 2022)). Background gene sets were set as the lists of PUFA genes presented in both the training and test cohorts separately for the classification of tumor and normal samples and for the molecular subtypes. Terms with adjusted *p*-value < 0.001 were considered statistically significant.

### 2.7. Differential Expression Analysis 

The expression levels of the genes that were selected as important features for dichotomic classification were compared between the tumor and normal sample groups with a two-sided Mann–Whitney test, followed by the Benjamini–Hochberg procedure for multiple comparisons. The false discovery rate (FDR) was set as 0.05. To find *PUFA* genes that were differentially expressed across molecular subtypes, the first one-way ANOVA test was applied to the expressions of the genes that were previously selected as important for the molecular subtype classification. Genes with significantly (adjusted *p*-value < 0.05) different expression means were further compared between subtypes with a one-sided Mann–Whitney test, followed by the Benjamini–Hochberg (FDR = 0.05). Tests were performed with the SciPy library (https://scipy.org/ (accessed on 18 February 2022)) and the statsmodel module in Python (https://www.statsmodels.org/stable/index.html (accessed on 18 February 2022)).

## 3. Results

### 3.1. Validation of Machine Learning Nonparametric Approach 

This study is based on the Random Forest (RF) machine learning approach. The RF model consists of a number of decision trees with thresholds learned from the training set. A direct combination of different datasets does not work in this framework due to the differences in platforms that do not allow us to learn the common thresholds even for similar biological conditions. This limitation was overcome by the combination of the nonparametric method with RF algorithm (see the Section 2.2). This approach allowed us to use merged datasets to learn and to test the RF model. The model was validated on a dichotomic classification of head and neck cancer and normal tissues based on the 1000 most variable genes (Supplementary Figure S1). Quality metrics (Balanced accuracy 0.99, ROC-AUC 0.99, PR-AUC 0.96) showed high performance and biological relevance of the most important features selected by Boruta [45] from the ranked expression levels of the 1000 genes.

### 3.2. Rank Model to Identify Most Important PUFA Genes for Breast Cancer vs. Normal Tissues Classification 

**Figure 1:**
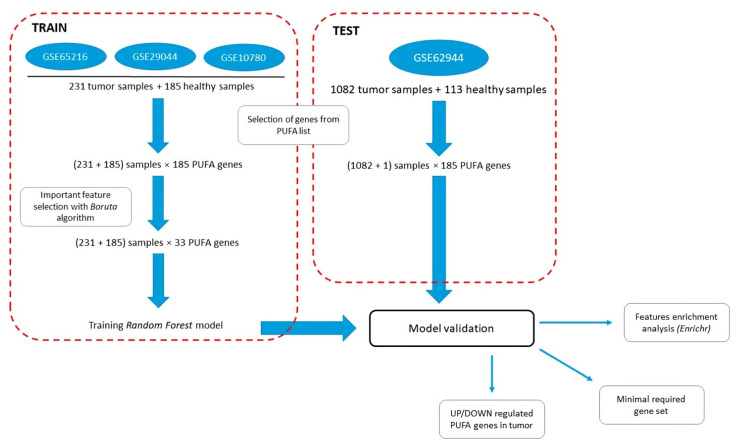
Workflow for studying differences in PUFA regulation between normal and breast cancer samples. Starting from the processed expression matrixes of the corresponding datasets taken from the GEO database, the training and test sets are constructed. To avoid the effect by platform differences, gene expression data within each sample are then converted to the ranks. PUFA genes are selected for the subsequent analysis (See details in the Section 2).

To assess the role of PUFA metabolism in the pathogenesis of BC, we compiled a list of 202 genes based on known data [13,14,53,54,55] that was previously described (Genes List in Supplementary Materials) [56]. We performed a systematic search for transcriptomes from open databases using previously developed tool ARGEOS [57], and we selected datasets GSE65216, GSE29044, GSE10780 (*n* = 231 tumor samples, *n* = 185 normal samples), and GSE62944 (*n* = 1082 tumor samples, *n* = 113 normal samples), with the latter representing TCGA data from the TCGA (The Cancer Genome Atlas) database. The datasets included samples of both breast cancer and normal adjacent tissues (Supplementary Table S2). Next, the initial list of PUFA genes selected overlapped with the genes presented in the datasets; 185 genes presented in all datasets were chosen for further analysis (Genes list, Supplementary). The chosen datasets were divided into two groups: training sets (GSE65216, GSE29044, and GSE10780) and test set (GSE62944). The workflow for further studying the differences in PUFA regulation between normal and breast cancer samples is presented in Figure 1.

We used our pipeline based on the Boruta feature selection method. We reran Boruta several times, and on each run, the method selected the genes with ranked expression levels that are reliably more important for the classification than their shuffled ranks; see the Section 2.3. for details. From the 185 PUFA genes, 33 genes (Supplementary Genes list) were chosen as important in the training set (Figure 1, left flowchart). 

**Figure 2:**
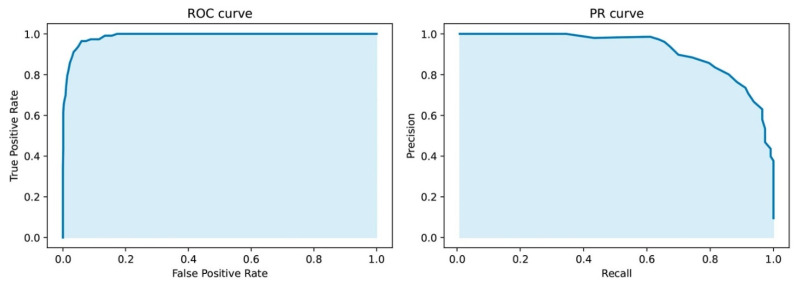
ROC and PR metrics for dichotomic classification of breast cancer samples and normal ones.

These genes were further used to learn a rank Random Forest dichotomic model. The model’s quality was evaluated on the test samples (Figure 1, right flowchart). The results are shown in Figure 2 and Supplementary Table S4.

The resulting classifier based on 33 *PUFA* genes effectively separates diseased and normal samples (Supplementary Table S4). This indicates that, indeed, the expression profiles of *PUFA* metabolism genes differ between normal and tumor tissues. 

Of the 33 selected genes, 6 genes were significantly (*p*-value < 0.05) upregulated (see Section 2.7) in the breast cancer samples and 24 genes were downregulated in comparison with normal tissues (Supplementary Table S5). To characterize these genes, an analysis of the GO functional and biological pathways, as well as the KEGG and WikiPathways pathways were performed using the Enrichr method (see the Section 2.6). KEGG enrichment indicated that the linoleic acid metabolic pathway was upregulated in breast cancer, while in normal adjacent tissues, arachidonic acid metabolic processes were the most enriched KEGG pathways (Supplementary Figures S3 and S4). Moreover, eicosanoid metabolism via the cyclooxygenase pathway was found to be downregulated in tumors compared with normal samples according to WikiPathways (Supplementary Figure S3). 

**Figure 3:**
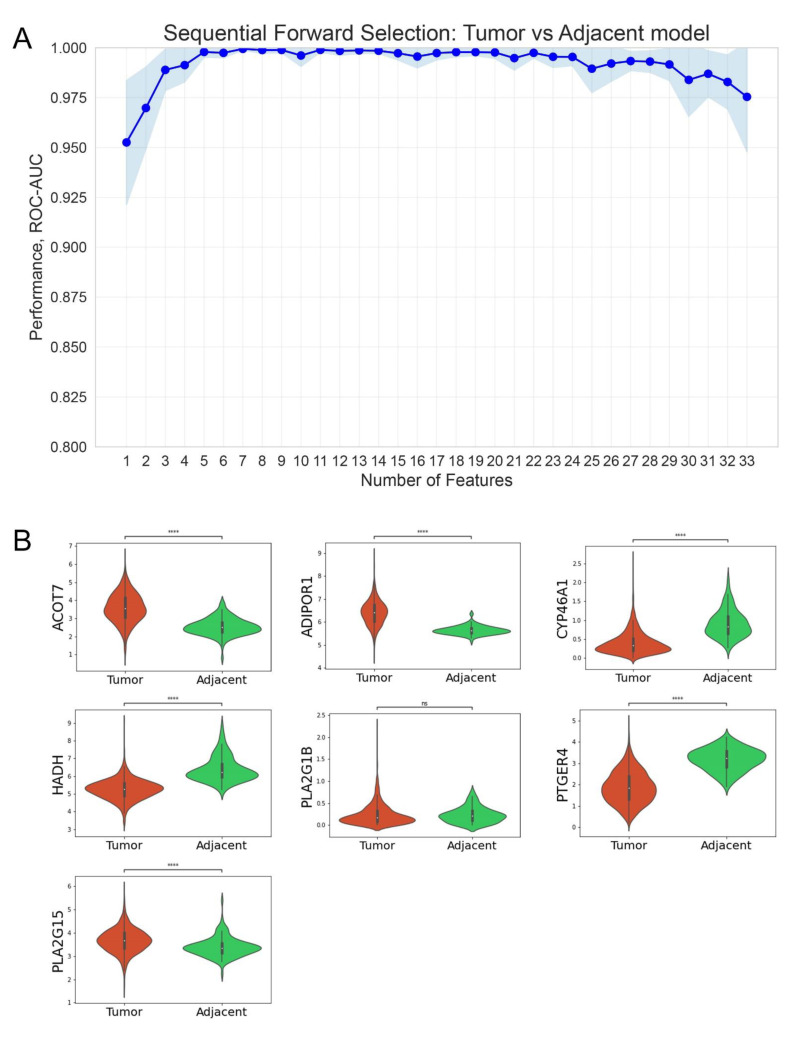
**** *p* < 0.0001. Identification of the minimal PUFA gene set required for efficient classification of breast cancer and normal samples. (**A**) Light blue zones refer to 95% confidence intervals. (**B**) Expression of *ADIPOR1*, *HADH*, *ACOT7*, *PTGER4*, *PLA2G15*, *PLA2G1B*, and *CYP46A1* genes in breast cancer and normal adjacent tissues.

At the next stage, we used the Sequential Feature Selector (SFS) method (see the Section 2.4) to identify the minimum set of genes that demonstrates the best quality of the tumor vs. normal tissue separation. The SFS algorithm has determined that the rank RF classifier based on a list of seven genes (ADIPOR1, HADH, ACOT7, PTGER4, PLA2G15, PLA2G1B, and CYP46A1) has the highest predictive efficiency according to ROC-AUC score (ROC-AUC 0.99, ci-bound 0.002) (Figure 3A). The expression of these genes in breast cancer and normal adjacent tissues is shown in Figure 3B.

### 3.3. Rank Model to Identify Most Important PUFA Genes for Breast Cancer Classification 

Breast cancer is a heterogeneous oncological disorder [58]. Since the emergence of high-throughput sequencing intrinsic molecular subtypes of breast cancer became widely used. Sørlie et al. distinguished five molecular subtypes: luminal A, luminal B, normal-like, HER2-enriched, and basal-like tumors [59]. These subgroups differ in prognosis and therapeutic strategies [60,61]. Thus, it is worth investigating the differences in PUFA metabolism not only between normal and cancer tissues but also between molecular subtypes. 

**Figure 4:**
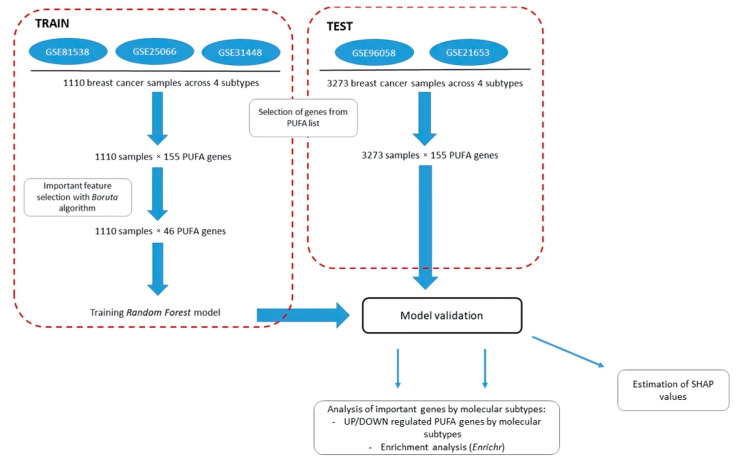
Workflow for studying differences in PUFA metabolism across four molecular subtypes of breast cancer. Processed expression matrixes for GSE81538, GSE25066, GSE31448, GSE21653, and GSE96058 datasets are collected from GEO database. Gene expression levels are ranked, and *PUFA* genes (155 genes) selected in training and test sets. Final model is built on the list of 46 important *PUFA* genes extracted by Boruta selection algorithm. (See details in the Section 2).

Aiming to address this question, we used five datasets from the GEO database: training sets (GSE81538, GSE25066, and GSE31448) and test sets (GSE96058 and GSE21653). The workflow for further studying the differences in PUFA regulation between tumor subtypes is presented in Figure 4. Due to the platform differences, only 155 genes from the full PUFA list (202 genes) were present in both sets and selected for further study (Supplementary Genes list). No normal-like subtype was considered due to the small number of samples presented in datasets. Our feature selection Boruta-based pipeline (see the Section 2.3.) marked 46 genes as important for separation of four molecular subtypes of breast cancer (Supplementary Genes list). Genes highlighted as important were further used for building the rank Random Forest classifier. The multi-class model had a balanced accuracy of 0.82 and an ROC-AUC of 0.85. As the test was not balanced between classes, it was worth looking at the quality metric for multi-class prediction F1-score, which was 0.75.

**Figure 5:**
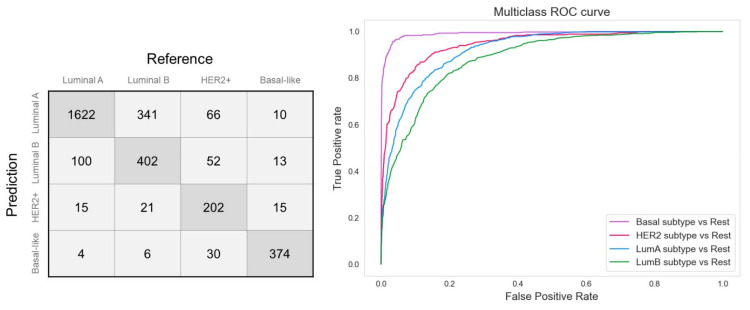
Confusion matrix for breast cancer molecular subtype predictor based on expression of PUFA genes.

The quality descriptors (balanced accuracy, ROC-AUC, and F1 score) of the constructed model show that the expression profiles of PUFA genes differ between the molecular subtypes. The largest number of misclassifications (Figure 5) falls on the luminal subtype (luminal A and luminal B) separation.

The 46 genes selected were analyzed to identify the subtype in which they are significantly (*p*-value < 0.05) differentially expressed. The analysis was carried out on the largest dataset (GSE96058) from the test set. Table 1 shows the genes in which the expression was significantly increased in the corresponding molecular subtype of cancer. It can be seen that for each subtype, a characteristic set of genes is revealed, most of them attributed to the group of genes responsible for ensuring the functioning of the signaling oxylipin system. The expression values for individual genes are shown in Supplementary Figure S5.

**Figure 6:**
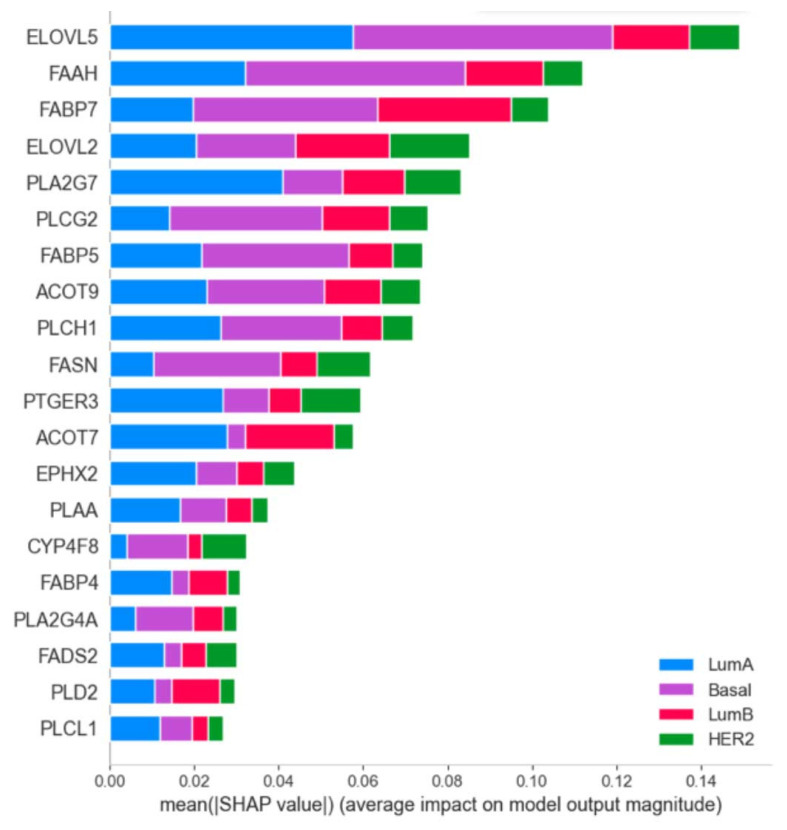
Most important features for classification between molecular subtypes of breast cancer. Importance was evaluated by SHAP values. Each color represents the importance of the separation of the corresponding class from the others.

We investigated the impact of the 46 genes utilized in the subtype classification using SHAP values (see the Section 2.5). The summary plot in Figure 6 shows the top 20 most influential genes. The color bar represents the features’ impact on separating the corresponding class from the others. ELOVL5 was the most important gene for overall classification, particularly, for the basal and luminal A subtypes. Rank FABP7 expression made the biggest impact on luminal B separation, while ELOVL2 expression made the biggest impact on the HER2-enriched subtype (Figure 6 and Figure S6).

## 4. Discussion

Here, we applied a rank Random Forest to the expression data of *PUFA* genes in BRCA to investigate their role in BRCA pathogenesis and in subtype phenotype differences. Our analysis shows that changes in the energy metabolism of PUFA, particularly, in the metabolism of signaling messenger oxylipins are important characteristics that can even be biomarkers for separating patients with BC from healthy people, as well as can determine the nature of molecular subtypes. The use of the feature selection RF-based algorithm Boruta made it possible to identify 33 PUFA metabolism genes that distinguish BC samples from normal tissues and 46 genes that differ between BC subtypes. 

It should be noted that the search for biomarkers (signatures) that allows for classifying breast cancer subtypes was carried out earlier (see, for example [62], where using copy number variant data can identify some biomarkers). The focus of our work was to evaluate the role of PUFA metabolic pathways in the biology of subtypes. The impetus for this study was previous work in which we compared the blood profile signatures of oxylipins and PUFAs in 152 healthy volunteers (HC) and 169 patients with various stages of BC [56]. Blood oxylipin signatures reflect the organism’s level of response to the disease. We also analyzed the DEGs of ten transcriptome datasets, and 19 genes for oxylipins biosynthesis were among the DEGs [56]. The SNP data for 33 genes related to oxylipin metabolism analysis reveal that CYP2C19, PTGS2, HPGD, and FAAH were on the list of DEGs in the analysis of transcriptomes and the list of SNPs associated with BC [56]. There is no doubt that PUFA metabolism is involved in BC manifestation, but further research is required to understand the mechanisms of interactions within PUFA metabolic cascades.

The rank RF model built on 33 selected genes showed high performance classifying breast cancer and normal adjacent tissues. The minimal number of genes required for the best performance (ROC-AUC 0.99, ci-bound 0.002; Figure 3) included seven genes (*ADIPOR1*, *HADH*, *ACOT7*, *PTGER4*, *PLA2G15*, *PLA2G1B*, and *CYP46A1*). In this list, *HADH* and *ACOT7* belong to the fatty acid beta-oxidation (FAO) pathway. Previously, for various malignant neoplasms, the so-called “lipolytic phenotype” was shown, in which the FAO pathway was reprogrammed [63,64]. Cancer cells can use changes in FAO metabolism for proliferation, survival, stemming, and metastasis [65]. The other four genes (*ADIPOR1*, *HADH*, *ACOT7*, *PTGER4*, *PLA2G15*, *PLA2G1B*, and *CYP46A1*) can be signed to the PUFA signaling function. 

Breast cancer is a highly heterogeneous disease; therefore, it is also essential to understand the diversity in PUFA metabolism across molecular subtypes for further research on the possibility of their use, both as a biomarker and target for therapy. We defined a list of 46 *PUFA* genes differentially across four molecular subtypes. Some of the genes identified in our study in “the distinguishing list” have previously been linked to cancer. It was shown that ELOVL5 (elongase, responsible for elongation of long chain fatty acids) is upregulated in breast cancer (BC) vs. normal adjacent tissue, with the expression correlated with changes in blood lipid species [66]. ELOVL2 expression was associated with malignant phenotypes and suggested as a novel prognostic biomarker in breast cancer [30]. On a cellular model, it was shown that ELOVL2 downregulation is associated with an increased likelihood of metastasis in breast cancer [30]. This is consistent with the data obtained suggesting that the level of ELOVL2 expression has the highest expression in the luminal A subtype, which has the best survival prognosis [4]. 

The *ACOT* (Acyl-CoA thioesterase) genes were mostly expressed in the basal-like and luminal B molecular subtypes. ACOT enzymes catalyze the hydrolysis of coenzyme A (CoA) esters to free fatty acids and CoA. Further pathways of these fatty acid’s metabolism are not completely clear. Additionally, acyl-CoA esters have more functions than simply an energy source, and modulation of their levels via ACOT enzymes activities is important for various pathways of lipid metabolism [67]. It was shown that an increased expression of ACOT1 was correlated with pivotal clinicopathological parameters and poor prognosis in gastric adenocarcinoma [68]. ACOT7 expression increased in lung and breast carcinoma, and low levels of its expression were associated with better survival prognosis [69]. This is also confirmed by the data obtained in the present work. A bar plot with the SHAP values shows the importance of the *ACOT7* gene expression for distinguishing luminal subtypes (Figure 6). Increased expression values of this gene are more likely to indicate the luminal B subtype, which is more aggressive than luminal A (Figure S6). 

Fatty acid-binding proteins (FABPs) are involved in binding, storing, and transporting to the appropriate compartments in the cell various fatty acids and other lipophilic ligands such as oxylipins and retinoids. This group of protein is tightly involved in inflammatory processes. Previous studies have revealed that FABP5 [70] and FABP7 [71] might regulate lipid quality and/or quantity to promote aggressiveness such as cell growth, invasiveness, survival, and inflammation in breast cancer cells. FABP7 was suggested as a potential target for the treatment complications of HER2 in breast cancer patients [71]. In our study, we found that a lower expression of this gene is the most important feature for determining luminal B subtype, while its higher expression levels make up the top five important features for basal-like breast cancer (Figure S6). FABP4 was also previously linked to the invasion and migration of colon cancer cells and obesity-associated breast cancer development [72,73]. We showed that the expressions of FABP4 are found in the luminal subtypes of breast cancer; nonetheless, it was not included in the most important hallmark of any subtype (Table 1 and Figure S6). 

Twenty of the most important features for classification between molecular subtypes of BC include genes that could be combined into groups of PUFA elongation or desaturation (ELOVL5, ELOVL2, and FADS2); intracellular transport (FABP4, FABP5, and FABP7); release of fatty acids from CoA esters (ACOT7 and ACOT9) and from more complex lipids (phospholipases PLA2G7, PLAA, PLA2G4A, PLCL1, PLCG2, PLCH1, and PLD2); and others, which include six genes attributed to various pathways *FASN* (fatty acid synthase catalyzes elongation of saturated fatty acids), *FAAH* (fatty acid amide hydrolase), *PTGER3* (prostaglandin EP3 receptor), *EPHX2* (soluble epoxide hydrolase), and *CYP4F8* (one of the monooxygenases that is specialized in the metabolism of PUFAs). The list of 185 genes took into account the processes of synthesis and degradation of fatty acids (both saturated and unsaturated), their transformation into oxylipins, and various oxylipin receptors. Interesting to note is that, besides *PTGER3* and *EPHX2*, all other genes from the list in Figure 6 can be attributed to processes that regulate the amounts and species of free fatty acids within cells. It is currently difficult to say why differences between these genes lead to differences in BC subtypes. All of the enzymes corresponding to these genes have been studied in different processes and have not previously been considered as a whole system. It is important that the study indicates the need for such a consideration.

## 5. Conclusions

Thus, BC subtypes can be discriminated by genes for fatty acid metabolism. A significant part of the genes that differ between subtypes refers specifically to the metabolism of PUFAs and regulatory oxylipins. This supposes that changes in PUFA metabolism are decisive in the manifestation of the subtype phenotypes. The use of rank RF has demonstrated the effectiveness of this approach and has yielded promising results. These results indicate that the genes found for FA metabolites may be potential biomarkers and therapeutic targets for different BC subtypes.

## Figures and Tables

**Table 1:** Genes that are upregulated in the respective subtype.

Luminal A	Luminal B	HER2+	Basal-Like
*ELOVL5*	*PTGES3*	FASN *	*AKR1B1*
ACAA1 *	*ADIPOR1*	*FABP6*	*CYP39A1*
*PLD2*	MBOAT7 *	*MGLL*	*PLD1*
ACAD8 *	ACOT8 *	*ALOX15B*	*PLA2G4A*
*PLCL1*	*CYP2B6*	*FADS2*	*FPR2*
*HPGDS*	*FAAH*		*PLCG2*
*CYP4F11*			*CYP7B1*
*PTGER3*			*FABP5*
*CYP4F8*			*PLA2G7*
*ELOVL2*			*CBR1*
*EPHX2*			*PLAA*
LPCAT3 *			ACOT9 *
*LTC4S*			*HSD17B12*
*FABP4*			*CYP39A1*
			*PLA2G2D*
			*PLCH1*

*—assigned to the group of genes responsible for the energy and structural functions of fatty acids; the rest can be attributed to the genes of the signaling oxylipin system.

## Data Availability

The data are available upon request.

## Supplementary Materials

The following supporting information can be downloaded at: https://www.mdpi.com/article/10.3390/cancers14194663/s1, Figure S1. Validation of the model was provided by the binary classification of head and neck cancer and healthy tissues based on 1000 most variable genes; Table S1. Genes_supplementary.xlsx; Table S2. Datasets selected for binary classification of healthy and tumor breast tissues; Table S3. Datasets selected for multiclass subtype classification of breast cancer; Table S4. Quality of rank RF for binary classification of healthy and tumor breast tissues; Table S5. Genes significantly upregulated in tumor samples (left column) and upregulated in healthy samples (right column); Figure S2. OOB error according to set number of trees (ntree) in the model for binary classification of normal and breast cancer tissues; Figure S3. Enrichment analysis of GO functional and biological pathways, as well as KEGG and WikiPathways pathways by genes important for classification of healthy and tumor breast tissues and upregulated in cancer samples; Figure S4. Enrichment analysis of GO functional and biological pathways, as well as KEGG and WikiPathways pathways by genes important for classification of healthy and tumor breast tissues and upregulated in healthy samples; Figure S5. Expression of FABP6, PLA2G7, ACOT7, FAAH, EPHX2 genes across subtypes; Figure S6. Top-5 most important genes for defining each of four molecular subtypes of breast cancer revealed by SHAP values.
