# Supplementary material for: Investigation of the Role of PUFA Metabolism in Breast Cancer Using a Rank-Based Random Forest Algorithm
Source: Cancers (Basel). 2022 Sep 25;14(19):4663. doi: 10.3390/cancers14194663 (PMC9562210; doi:10.3390/cancers14194663)
Supplement: Supplementary file 1 [file cancers-14-04663-s001.zip › cancers-1833364-supplementary.pdf]

# Supplementary
# Investigation of the Role of PUFA Metabolism in Breast Cancer Using Rank-Based Random Forest Algorithm

*Mariia V. Guryleva[1], Dmitry D. Penzar[1,2], Dmitry V. Chistyakov[3,*], Andrey A. Mironov [3,4], Alexander V. Favorov[2,5], and Marina G. Sergeeva[3]*

1 Faculty of Bioengineering and Bioinformatics, Lomonosov Moscow State University, 119234 Moscow, Russia
2 Vavilov Institute of General Genetics, Russian Academy of Sciences, Moscow, Russia
3 Belozersky Institute of Physico-Chemical Biology, Lomonosov Moscow State University, 119992 Moscow, Russia
4 Kharkevich Institute of Information Transmission Problems, Russian Academy of Sciences, Moscow, 127051 Russia
5 School of Medicine, Johns Hopkins University, Baltimore, MD, USA
* Correspondence: chistyakov@belozersky.msu.ru; Tel.: +7-495-939-4332

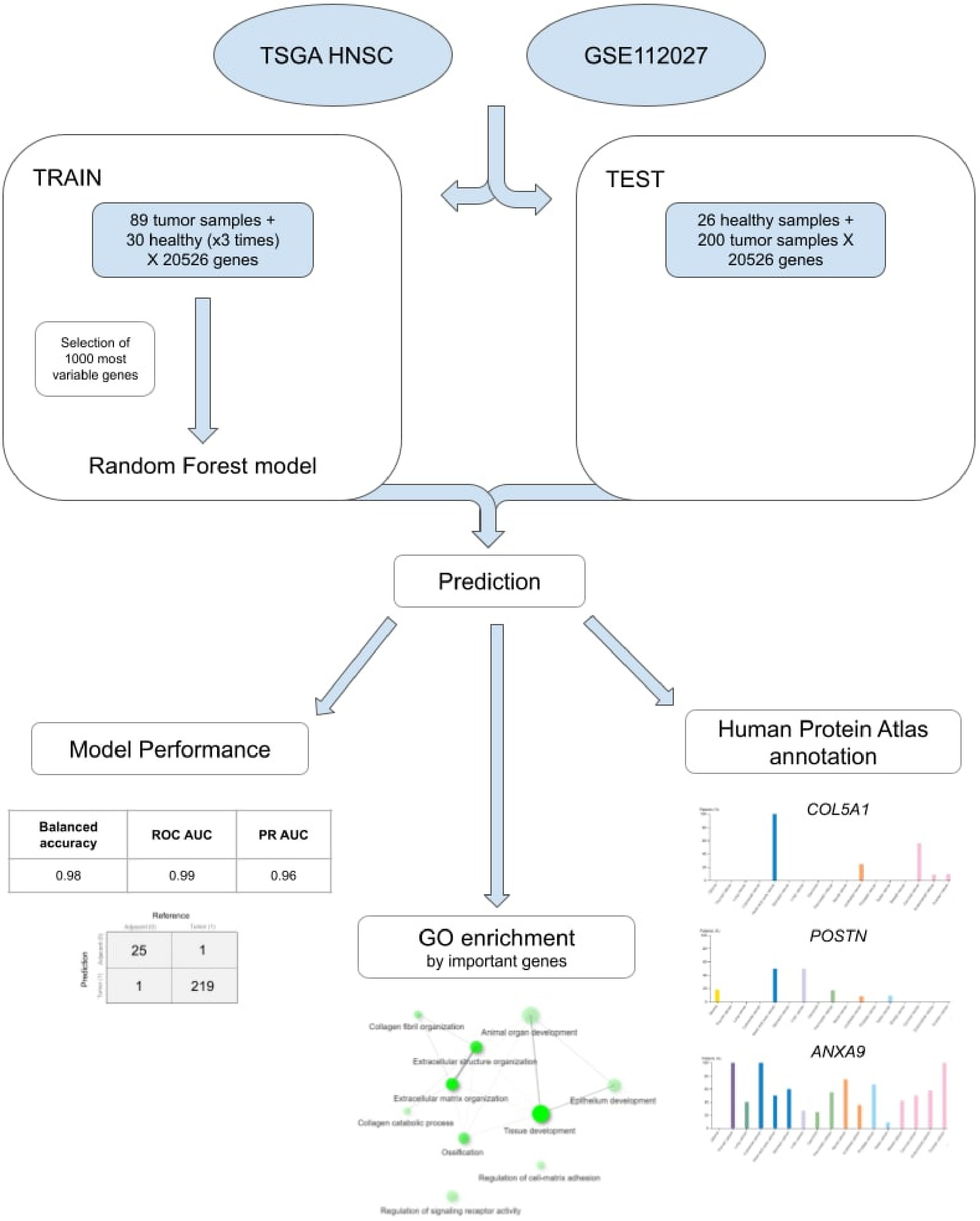


**Figure S1.** Validation of the model was provided by the binary classification of head and neck cancer and healthy tissues based on 1000 most variable genes.

**Table S1. Genes_supplementary.xlsx.**
Column 'Tumor_vs_Healthy' - genes used for binary classification of healthy and tumor breast tissues. 17 genes from the PUFA list were not considered as they were not evaluated in the platforms from the train or test sets.
Columns 'Important_genes_Tumor_vs_Healthy' - genes selected by *Boruta* algorithm as important for classification of healthy and breast cancer samples.
Column 'Molecular_subtypes' - genes used for classification of molecular subtypes. 47 genes from the PUFA list were not considered as they were not evaluated on the platforms from the train or test sets.
Columns 'Important_genes_Molecular_subtypes' - genes selected by *Boruta* algorithm as important for classification of breast cancer molecular subtypes.

**Table S2.** Datasets selected for binary classification of healthy and tumor breast tissues.

| GEO ID | Platform | Tumor samples | Healthy samples | TRAIN/TEST |
|---|---|---|---|---|
| GSE65216 | GPL570 | 153 | 11 | TRAIN |
| GSE29044 | GPL570 | 78 | 31 | TRAIN |
| GSE10780 | GPL570 | - | 143 | TRAIN |
| GSE62944 (TCGA) | GPL9052 | 1082 | 113 | TEST |

**Table S3.** Datasets selected for multiclass subtype classification of breast cancer.

| GEO ID | Platform | Total number of samples | TRAIN/TEST |
|---|---|---|---|
| GSE81538 | GPL11154 | 383 | TRAIN |
| GSE25066 | GPL96 | 464 | TRAIN |
| GSE31448 | GPL570 | 263 | TRAIN |
| GSE96058 | GPL11154 | 3052 | TEST |
| GSE21653 | GPL570 | 221 | TEST |

| GEO ID | Luminal A | Luminal B | HER2-enriched | Basal-like |
|---|---|---|---|---|

| | | | | |
|---|---|---|---|---|
| GSE81538 | 156 | 105 | 65 | 57 |
| GSE25066 | 160 | 78 | 37 | 189 |
| GSE31448 | 90 | 49 | 26 | 98 |
| GSE96058 | 1657 | 729 | 327 | 339 |
| GSE21653 | 84 | 41 | 23 | 73 |

**Table S4.** Quality of rank RF for binary classification of healthy and tumor breast tissues. Confusion matrix and quality metrics scores are shown.

| Prediction \ Reference | Tumor | Adjacent |
|---|---|---|
| Tumor | 1037 | 9 |
| Adjacent | 45 | 104 |

| | Balanced accuracy | ROC AUC | PR AUC |
|---|---|---|---|
| **Rank-model** | 0.9394 | 0.9889 | 0.9119 |

**Table S5.** Genes significantly upregulated in tumor samples (left column) and upregulated in healthy samples (right column).

| Tumor samples |
|---|

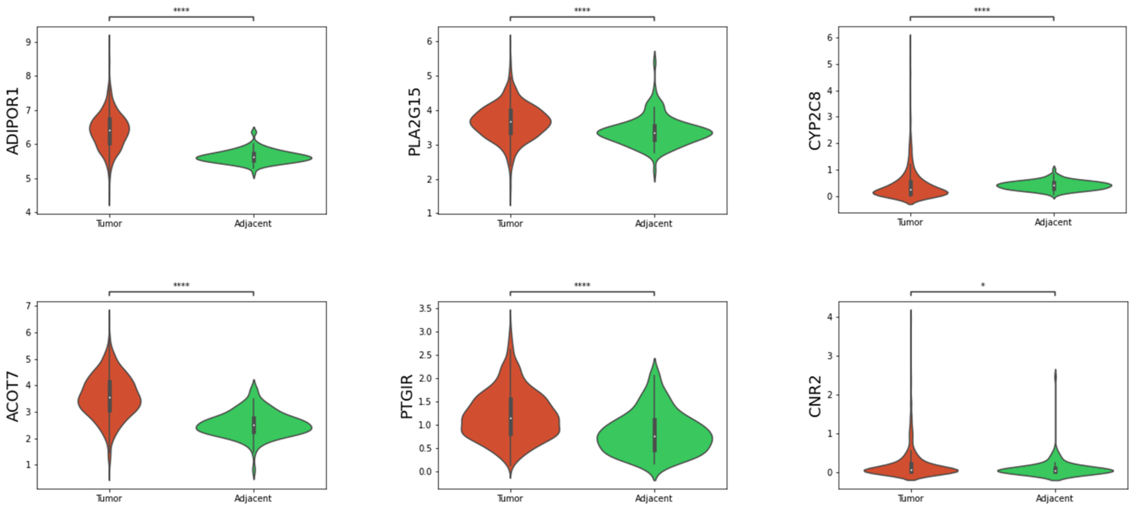

## Healthy samples

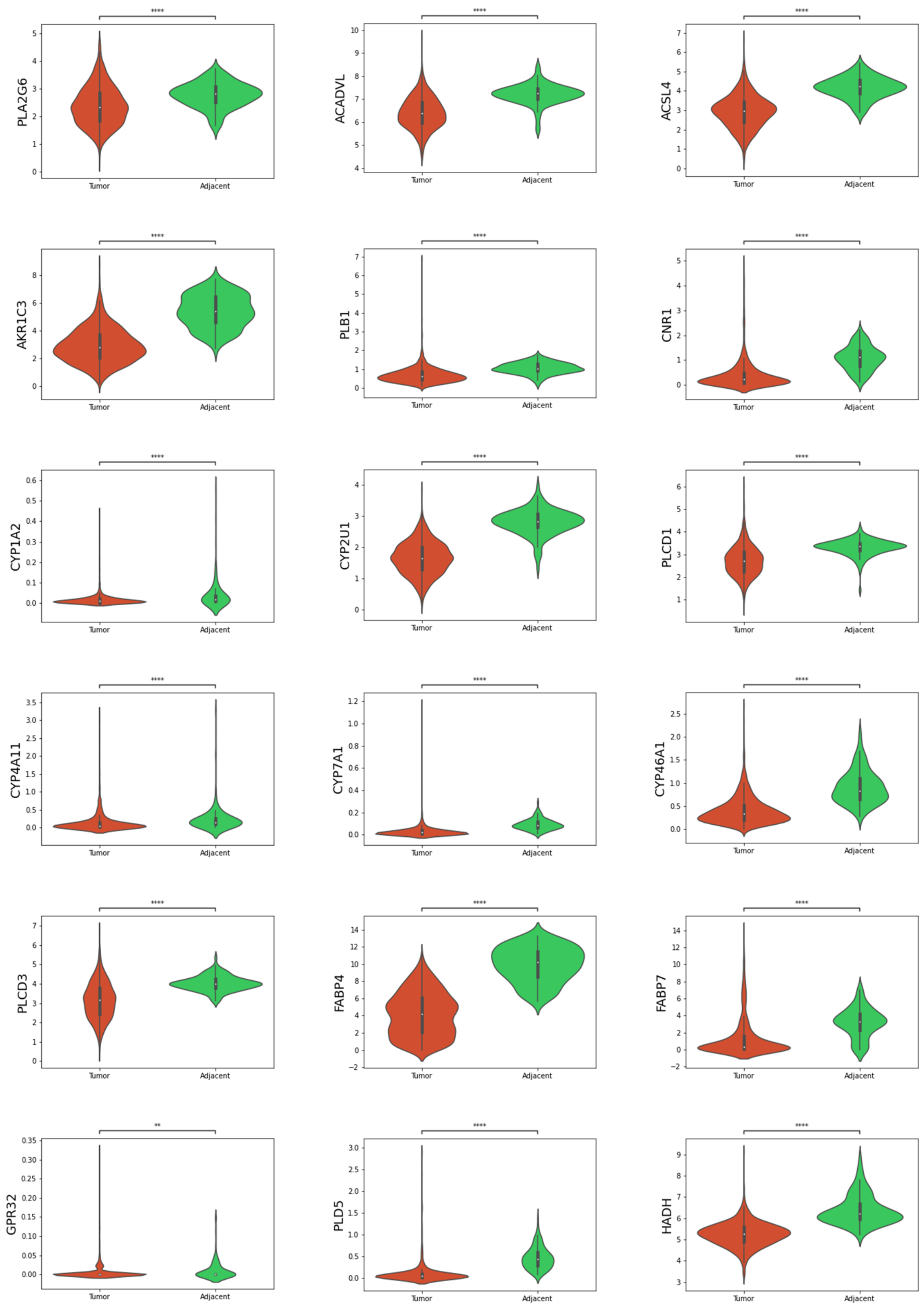

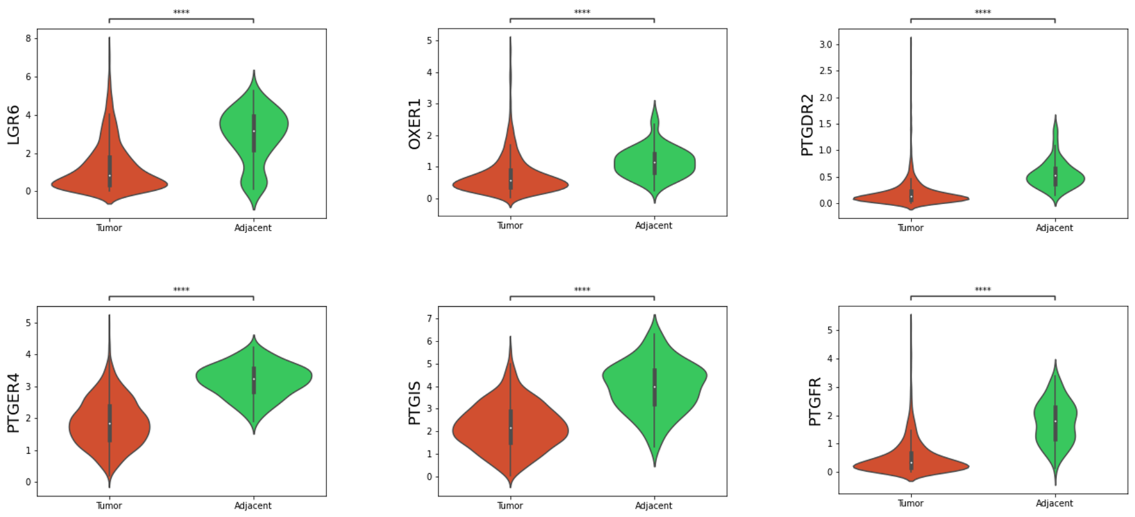


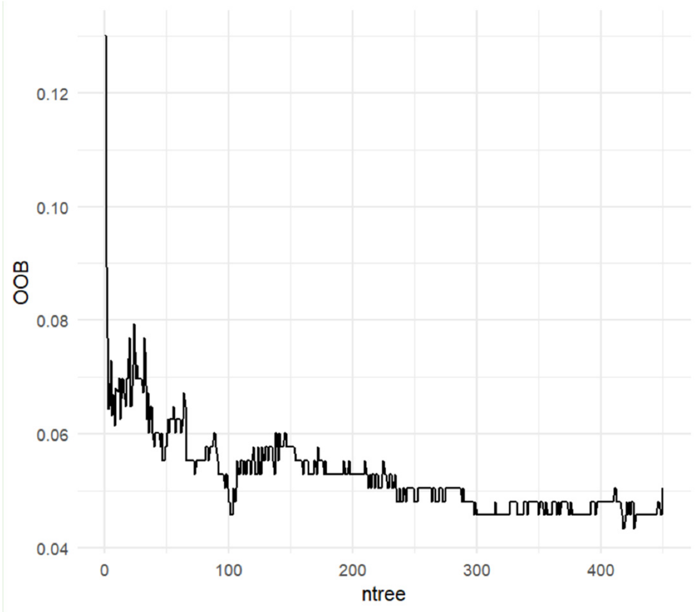


**Figure S2.** OOB error according to set number of trees (ntree) in the model for binary classification of normal and breast cancer tissues.

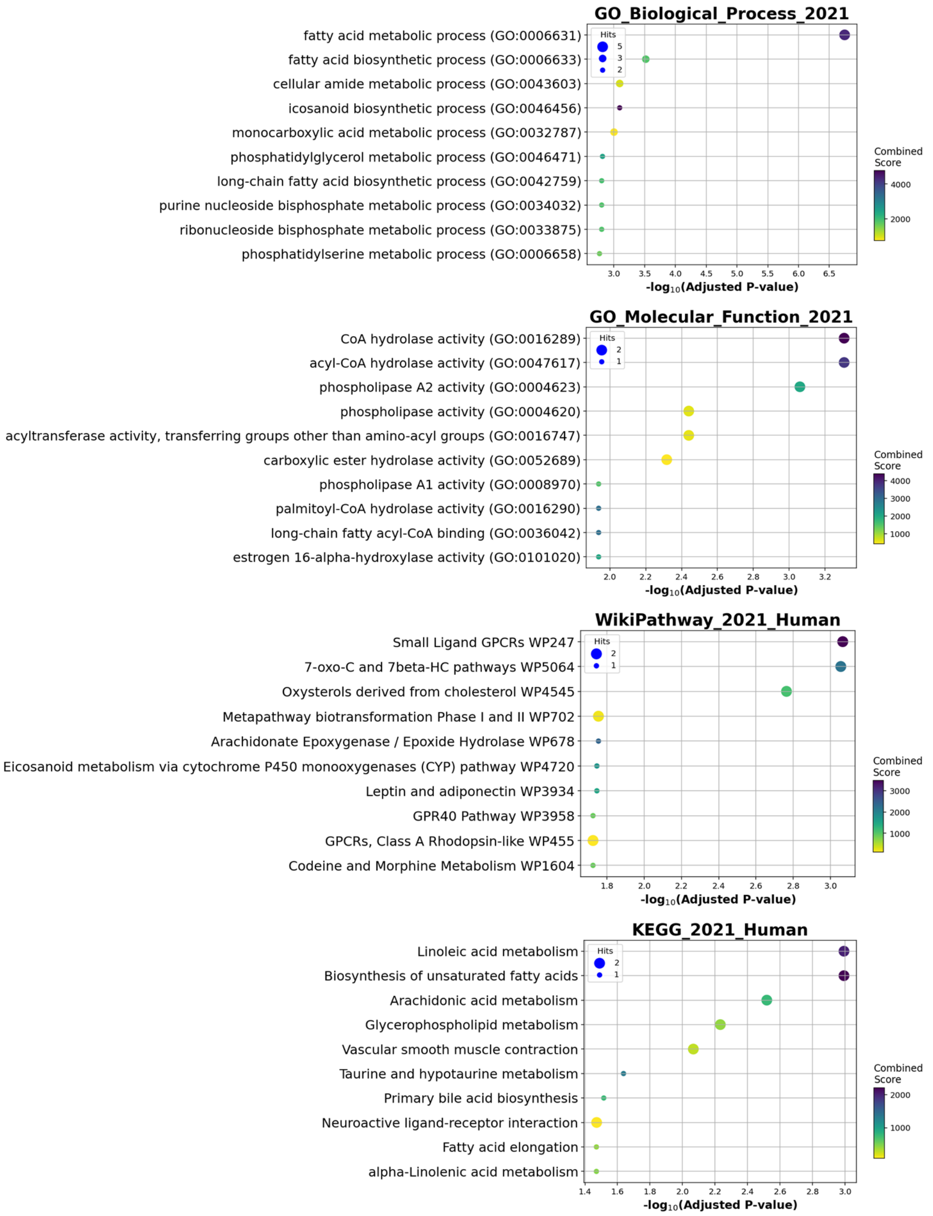


**Figure S3**. Enrichment analysis of GO functional and biological pathways, as well as KEGG and WikiPathways pathways by genes important for classification of healthy and tumor breast tissues and upregulated in cancer samples.

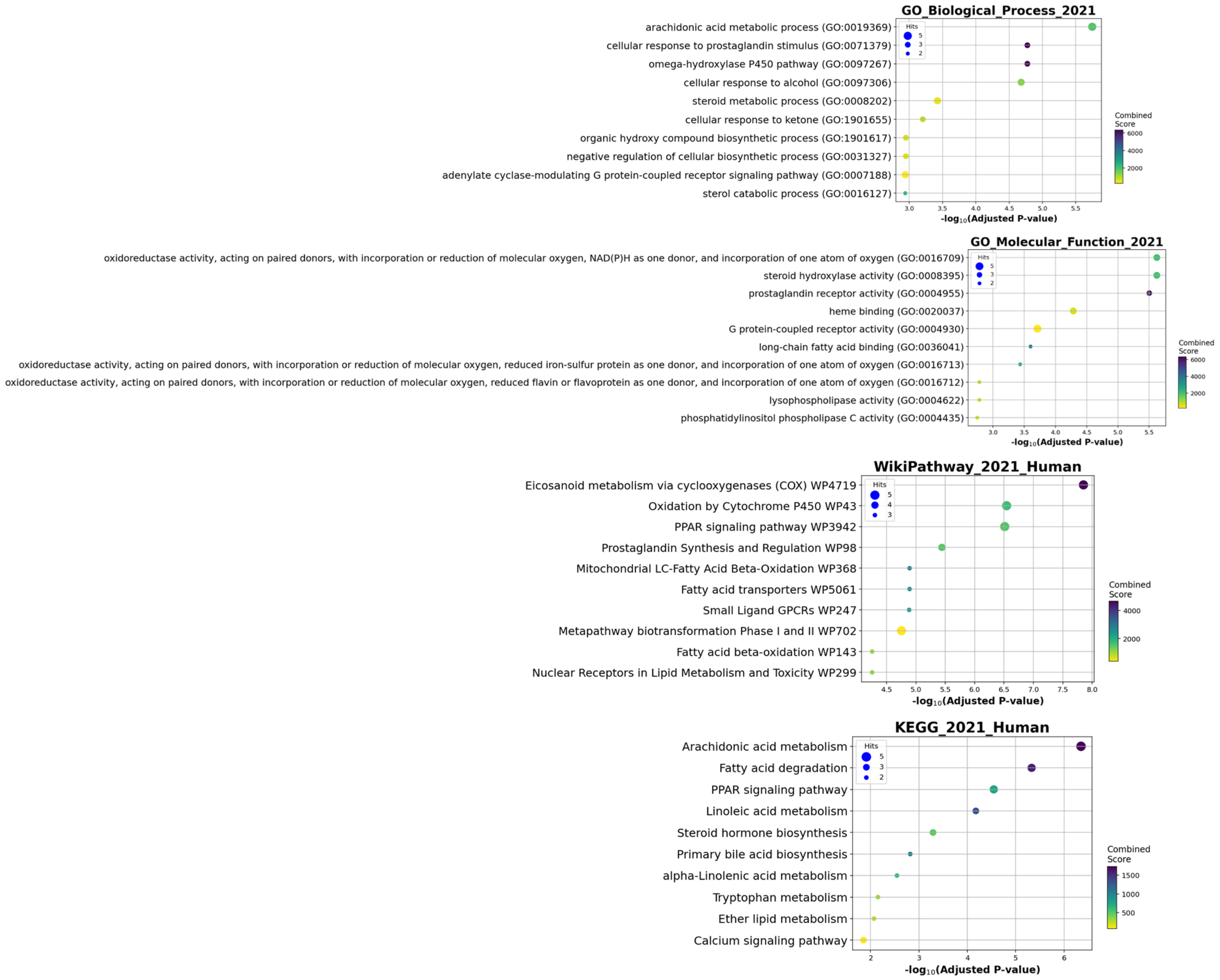


**Figure S4**. Enrichment analysis of GO functional and biological pathways, as well as KEGG and WikiPathways pathways by genes important for classification of healthy and tumor breast tissues and upregulated in healthy samples.

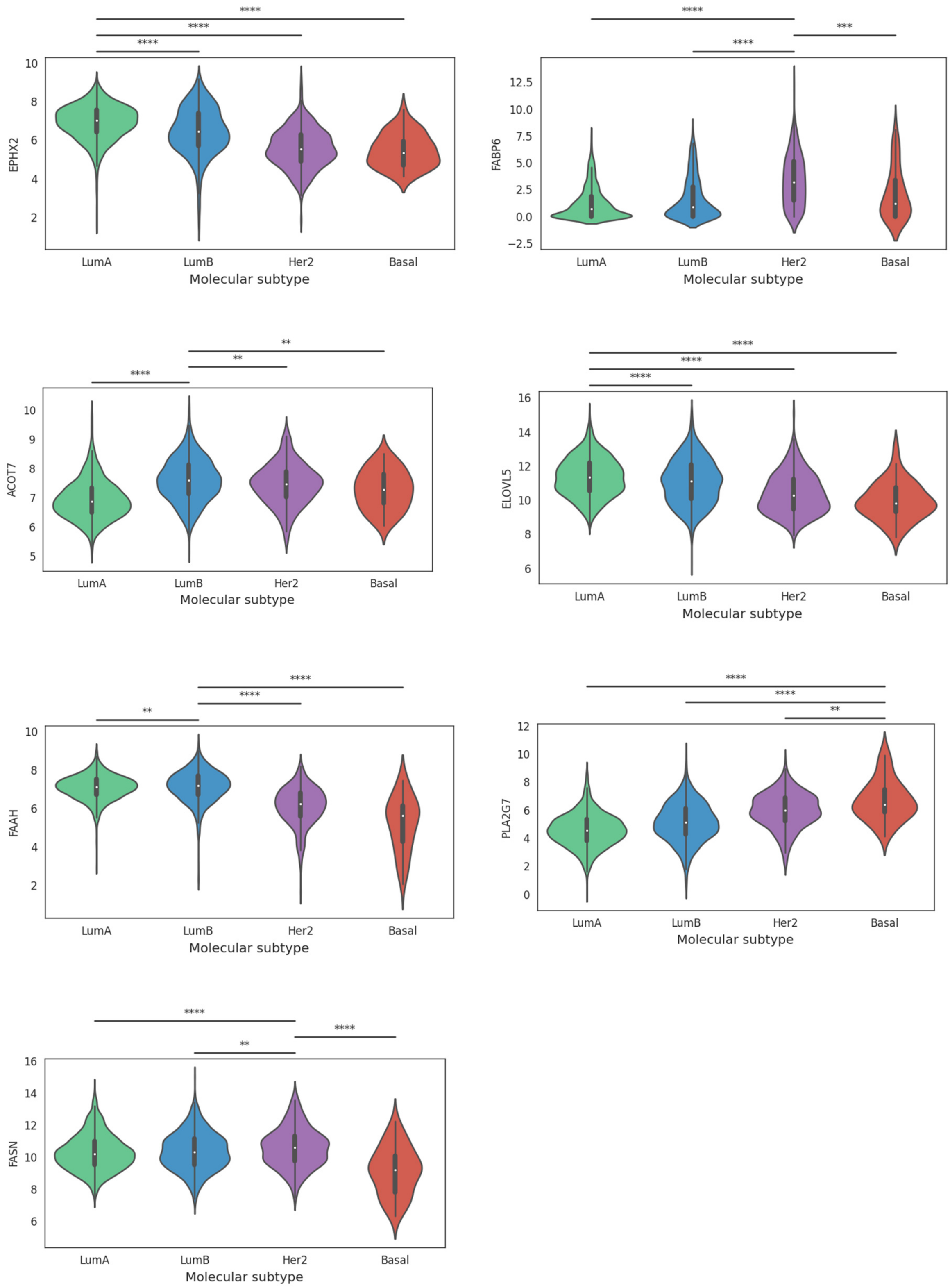


**Figure S5.** Expression of FABP6, PLA2G7, ACOT7, FAAH, EPHX2 genes across subtypes.

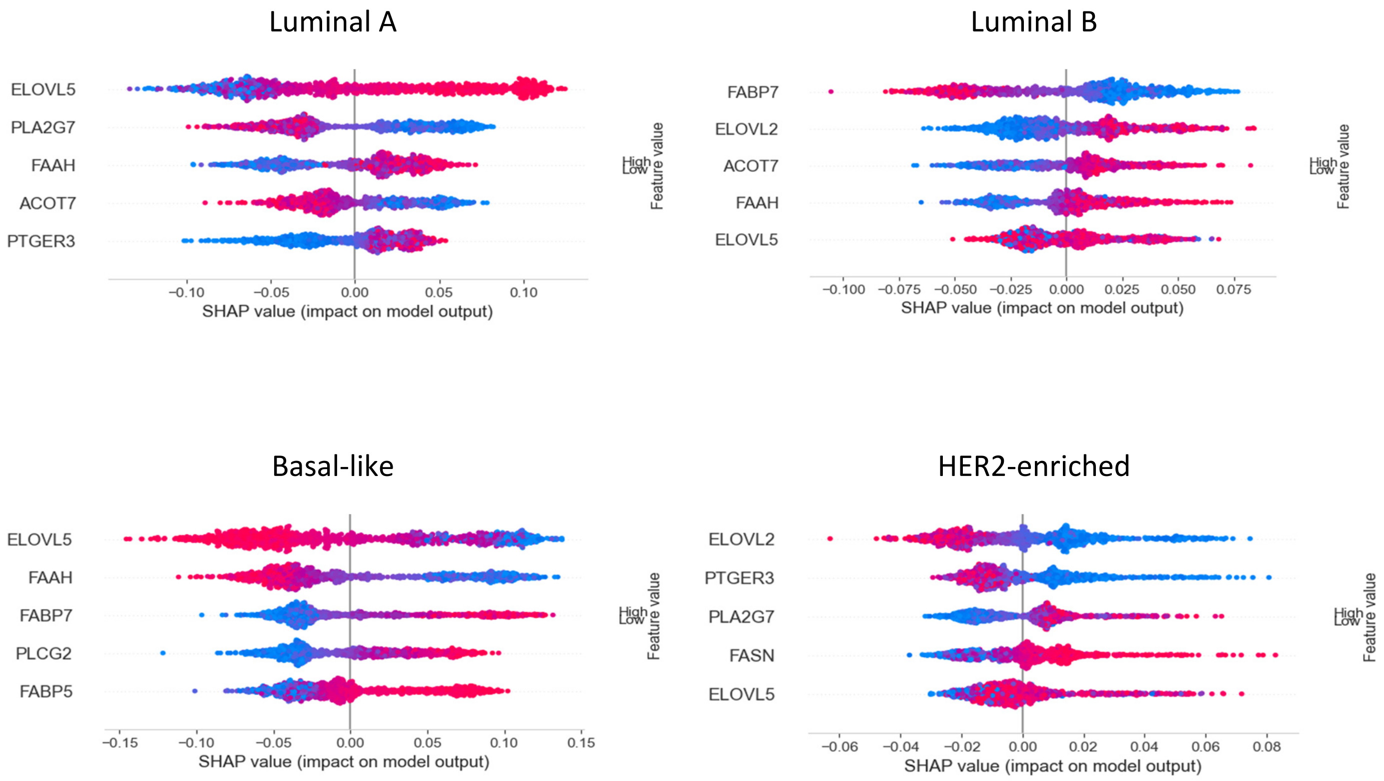


**Figure S6.** Top-5 most important genes for defining each of four molecular subtypes of breast cancer revealed by SHAP values. The dot represents the importance in each sample. Blue color refers to lower expression loevel, while red - to higher.
